# From Pathology to Materials Science and Engineering: Harnessing the Amyloid State for Biotechnological Applications

**DOI:** 10.1021/acsami.5c11847

**Published:** 2025-11-10

**Authors:** Lucas B. Fallot, Chandramouli Natarajan, Carol A. Anderson, Enoch A. Nagelli, F. John Burpo, Ryan Limbocker

**Affiliations:** † Department of Chemical and Biological Science and Engineering, 8531 United States Military Academy, West Point, New York 10996, United States; ‡ Photonics Research Center, United States Military Academy, West Point, New York 10996, United States

**Keywords:** biophysics, drug delivery, tissue engineering, antimicrobials, purification, detection, catalysis, bioelectronics

## Abstract

The aberrant misfolding and aggregation process of specific peptides and proteins plays a seminal role in the onset and development of over 60 protein misfolding diseases, including Alzheimer’s and Parkinson’s diseases. These proteins can convert from the endogenous, monomeric, and often largely intrinsically disordered state to the pathological, highly ordered amyloid state, which results in the formation of long, thread-like fibrillar species with extensive β-sheet structure and hallmark tinctorial and biophysical properties. Beyond pathology, the amyloid state has been well-studied for its role in physiological processes in numerous organisms through functional amyloids. In this review, we consider principles governing amyloid formation, with a focus on leveraging the unique biophysical properties and templating abilities of amyloids to produce diverse amyloid-containing materials with wide-ranging biotechnological applications, including, but not limited to, aerogels and hydrogels of varied function, drug delivery, tissue engineering, antimicrobials, purification and detection, protein-based packaging and food science, chemical catalysis, and bioelectronics. We conclude with a brief discussion on the opportunities and challenges ahead for implementing amyloid-based biotechnologies in society.

## Graphical abstract

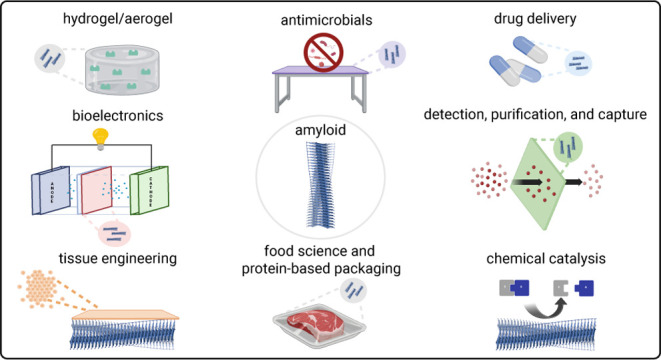

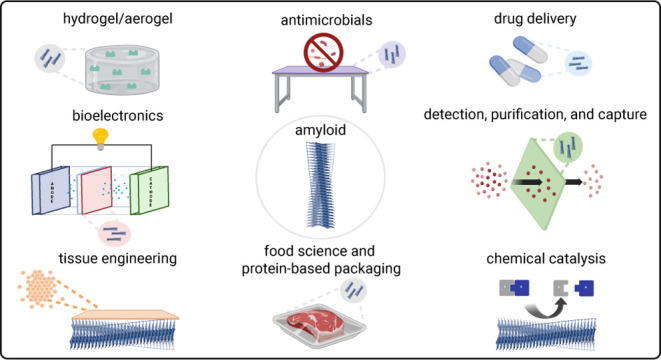

## 1 Introduction

The self-assembly of soluble, monomeric peptides and proteins into intractable, insoluble amyloid aggregates remains an area of intense study.^1–3^ In human disease, amyloids form following the misfolding and aggregation of these biomolecules into remarkably stable, thread-like fibrillar species with a signature cross-β core comprised of β-strands arranged perpendicular to the fibril axis (Figure 1).^2^ Amyloids are generated through nanoscale processes that are dynamical and complex; their mechanism of formation can vary considerably depending upon the protein of interest, as well as the solution conditions for in vitro assays and cellular microenvironment for in vivo processes.^1^ Relevant intrinsic and extrinsic factors that influence fibril formation include temperature, pH, ionic strength, mutation to the protein sequence, the introduction of surfaces for heterogeneous nucleation, the presence of specific free lipids or aggregated lipids in membranes, destabilizing solvents, denaturants or chaotropic agents, shaking in vitro and seemingly traumatic brain injury in vivo, and post-translational modification.^1,4–8^ Of further note for lipids, amyloid fibril deposits from multiple diseases have been observed to be enriched in common lipidic components, including cholesterol and sphingolipids that are constituents of lipid rafts,^9^ and specific compositions of lipid vesicles are known for their ability to promote the nucleation of amyloidogenic proteins.^10^

**Figure 1:**
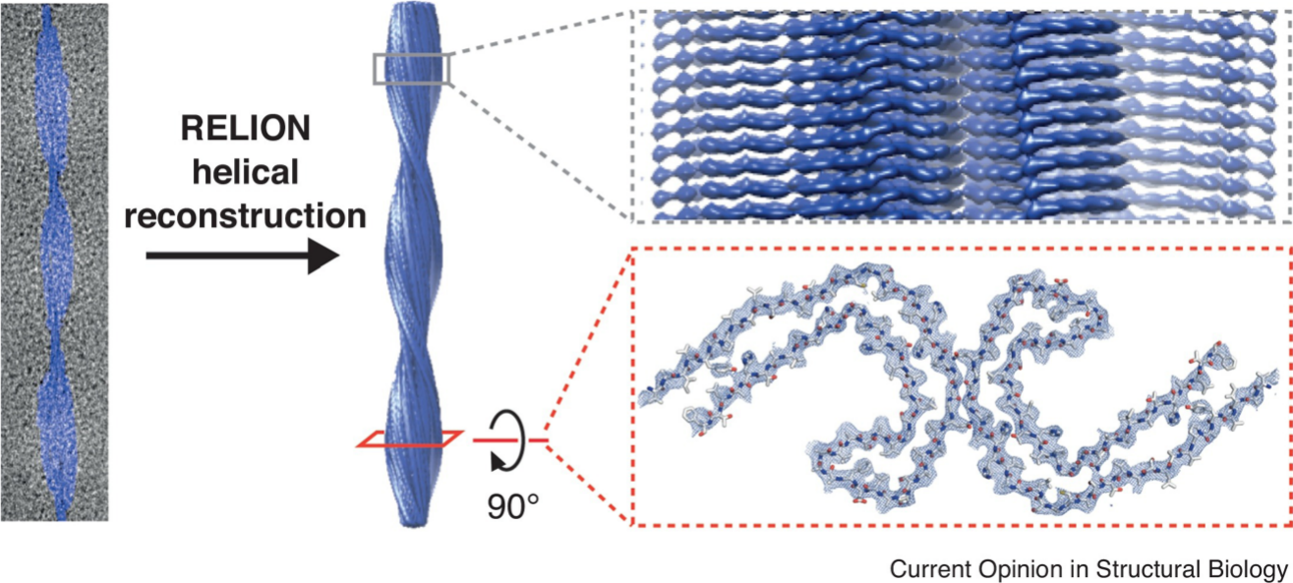
Atomic structure determination process for tau paired helical filaments isolated from post-mortem Alzheimer’s disease brains and imaged by cryogenic electron microscopy (cryo-EM). Analysis of the 3D reconstructed paired helical filament structure (middle) shows individual β-strands along the helical axis (top right, grey box) and side chains of amino acid residues in the filament core in the plane perpendicular to the fibril axis (bottom right, red box) formed of eight β-stands arranged to form a double-layered C-shape in the Alzheimer tau fold. Reproduced with permission from ref 13 Copyright 2020, Elsevier.

Extensive evidence supports that the sequence of a protein and its environment are highly relevant to disease and can result in drastic differences in the thermodynamics and kinetics regulating amyloid fibril formation, ultimately producing aggregates with unique biophysical properties and differential abilities to template further protein self-assembly through protein-specific processes that can enable passive self-replication or seed amplification.^1^ Alongside numerous examples of engineered amyloids for biotechnological applications that are the focus of this review, these observations highlight the potential for tuning protein sequence and solution conditions to control the fundamental kinetic and thermodynamic parameters governing aggregation, leading to the cost- and time-effective generation of rationally designed amyloids with diverse material properties and applications.

While the importance of the amyloid state in pathology has been clear for decades following the observation by electron microscopy in 1959 that ultrathin sections of amyloidotic tissues contained fibrillar aggregates,^11^ only more recently did applications of amyloids for materials science purposes become a central area of investigation around the globe. This research has undergone rapid growth over the past decade (Figure 2). Considering that highly diverse protein sequences can be driven toward the amyloid state under the right (or wrong, in the context of pathology) conditions and existing applications of amyloids, we anticipate the continued design of presently uncharacterized amyloids and amyloid-containing materials from differing monomeric peptides and proteins, as well as the optimization of existing ones.

**Figure 2:**
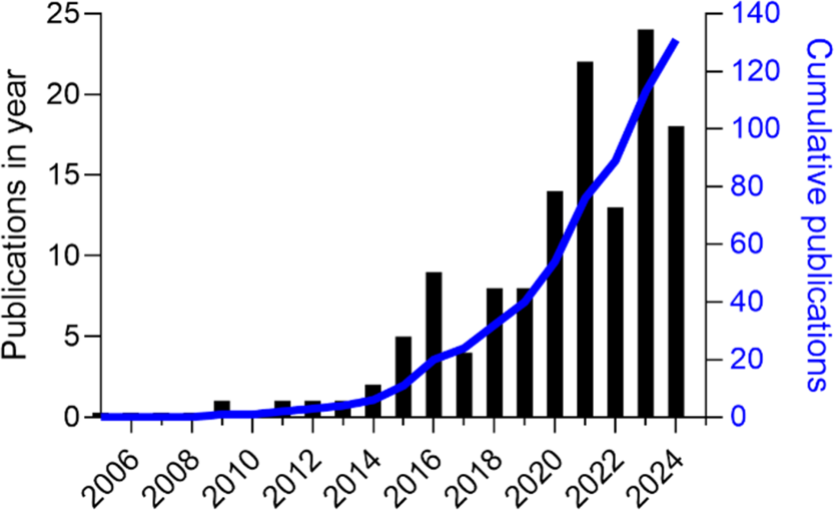
Number of publications in ACS Applied Materials and Interfaces containing “amyloid” in the abstract. Substantial progress has been made in the field leveraging amyloids for materials science purposes. Left *y*-axis (black bars) shows publications in a given year from 2006 to 2024. Right *y*-axis (blue line) shows the cumulative total number of publications over this period.

We begin this review focusing on the morphological and physicochemical properties of amyloid fibrils and mechanisms by which their templated growth and proliferation can occur, to highlight the kinetic, thermodynamic, and biophysical principles underpinning amyloid formation (Section 1). We then review salient applications demonstrating the use of amyloids for materials science purposes, therein harnessing the amyloid state’s tensile strength, mechanical properties, potential for biocompatibility and sustainability, and templating abilities for the production of diverse materials with wide-ranging biotechnological applications (Section 2). These applications include but are not limited to biosensing, purification, diverse hydrogels and aerogels, chemical catalysis for targeted molecular degradation relevant to both pathology and materials science, tissue engineering, bioelectronics, and antimicrobials. We conclude with a critical look at how amyloids may be used in the future to expand the above areas of interest, with consideration of factors to achieve practical benefit for society (Section 3).

### 1.1 Pathological and Functional Amyloids

Discrete proteins aggregate into hallmark fibrillar aggregates in (1) neurodegenerative diseases, such as the amyloid-β peptide (Aβ) and tau protein in Alzheimer’s disease (AD), the α-synuclein protein in Parkinson’s disease (PD), and huntingtin fragments in Huntington’s disease; (2) non-neuropathic systemic amyloidoses, such as immunoglobin light chains or fragments thereof in amyloid light chain amyloidosis and lysozyme mutants in lysozyme amyloidosis; and (3) non-neuropathic localized amyloidoses in specific tissues, such as apolipoprotein A1 fragments in Apolipoprotein A1 amyloidosis and islet amyloid polypeptide (amylin) in Type II diabetes (Table 1).^3^ The great diversity in the length and composition of protein sequences for the above proteins emphasizes that many proteins can enter the amyloid state under specific conditions, and it is often considered that the cross-β fold may be an intrinsic feature of a polypeptide chain.^12^ Despite precursor amyloidogenic proteins lacking sequence similarity, which can vary dramatically in their sequence lengths and the degree to which their monomeric form is natively unfolded, folded, or even a protein assembly, the macroscopic features of amyloid fibrils are conserved.^12^

**Table 1:** Examples of Peptides and Proteins That can Enter the Amyloid State, Highlighting Their Diverse Functions^a^

peptide or protein	classification	relevance
fragments of the amyloid-β peptide (particularly Aβ40 and Aβ42)	pathological (neurodegenerative)	Alzheimer’s disease (AD)
Tau		AD, Pick’s disease, chronic traumatic encephalopathy (CTE), corticobasal degeneration, and progressive supranuclear palsy
α-synuclein		Parkinson’s disease (PD), dementia with Lewy bodies, PD dementia, multiple system atrophy
fragments of huntingtin protein		Huntington’s disease
superoxide dismutase 1		amyotrophic lateral sclerosis
transthyretin		familial amyloidotic polyneuropathy
prion protein		spongiform encephalopathies
		
immunoglobulin light chains and its fragments	pathological (non-neuropathic systemic amyloidosis)	amyloid light chain amyloidosis
serum amyloid A1 fragments		amyloid A amyloidosis
lysozyme mutants		lysozyme amyloidosis
		
apolipoprotein A1 fragments	pathological (non-neuropathic localized amyloidosis)	apolipoprotein A1 amyloidosis
amylin		type II diabetes
		
hormones	functional (storage)	stored as secretory granules
		
curli	functional (structure)	biofilm formation
chorion		structural protection from environmental toxins
Orb2		maintenance and recall of memory in flies
		
Sup35	functional (loss-of-function)	translation termination factor in yeast
	
Cdc19		pyruvate kinase in yeast; reversible aggregation triggered by stress
		
Het-s	functional (signaling/gain-of-function)	membrane penetration
RIP1/RIP3 amyloid system		necroptosis
		
synthetic, engineered, or naturally ocurring amyloidogenic proteins	biotechnological applications	see Section 2

a For additional examples, see refs 1–3 for pathological amyloids, ref 15 for functional amyloids, and Section 2 herein for biotechnological applications of amyloids.

Despite possessing conserved macroscopic features and hallmarked properties, the atomic structures of fibrils can vary considerably depending on the amyloidogenic protein undergoing fibril formation. This has been shown by cryo-EM-derived atomic structures for fibrils of Aβ40 ^184^ (Figure 3a) and Aβ42 in AD^185^ (Figure 3b), tau in AD^186^ (Figure 3c), α-synuclein in PD, Parkinson’s disease dementia (PDD), and Dementia with Lewy Bodies (DLB)^187^ (Figure 3d), Serum amyloid A1 in vascular AA amyloidosis^188^ (Figure 3e), and transthyretin in hereditary Val30Met ATTR amyloidosis^189^ (Figure 3f). Functional amyloids also have diverse atomic fibril structures, as shown similarly for FapC from *Pseudomonas* sp. UK4 ^121^ (Figure 3g), receptor-interacting protein kinases 3 (RIPK3) relevant to necrotic pathways in humans^190^ (Figure 3h), the synaptic translation regulator Orb2 involved in *Drosophila* memory^18^ (Figure 3i), and uperin 3.5 (Figure 3j) and aurein 3.3 (Figure 3k) involved in amphibian antimicrobial responses.^120^ Moreover, fibrils formed from the same protein can be polymorphic. The tau protein, for example, forms fibrils with core structures that can have pathology-specific folds, including in AD, Pick’s disease, chronic traumatic encephalopathy, corticobasal degeneration, progressive supranuclear palsy, and others. Polymorphisms have also been observed for fibrils of Aβ42 (Figure 3b), where Type I filaments were found mostly in sporadic AD and type II filaments were found in familial AD and other conditions.^185^ Indeed, multiple filament types are often observed in a single disease (Figure 3b,c). These findings highlight the importance of solution conditions in the formation of pathological amyloids, emphasized further by a recent study showing that in vitro assembly conditions with recombinant tau can drive the formation of vastly different amyloid fibril polymorphs, a small subset of which recapitulated the AD and chronic traumatic encephalopathy (CTE) fibril fold when inorganic salt concentration and shaking were carefully controlled.^14^

**Figure 3:**
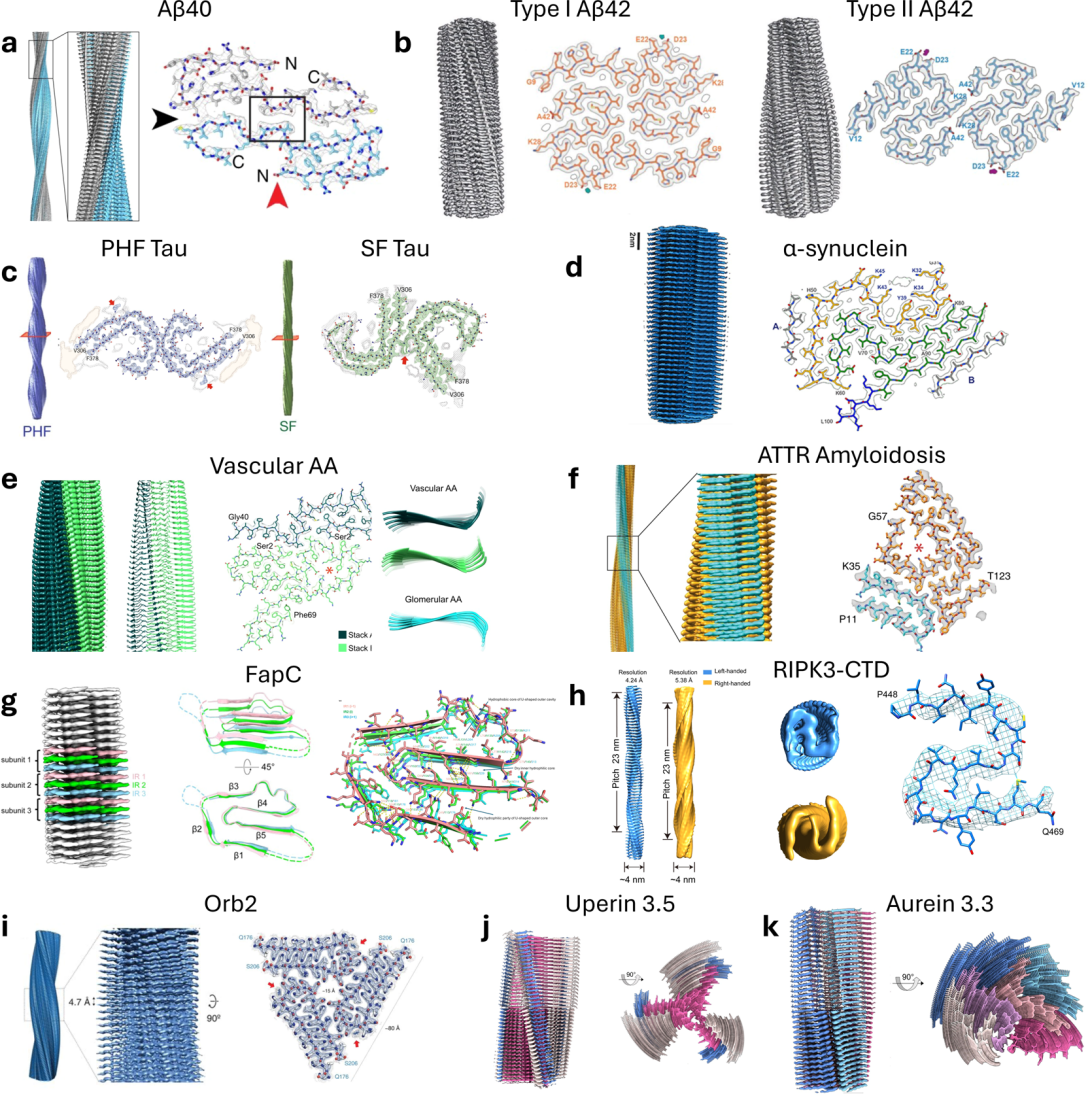
Cryo-EM-derived atomic structures of pathological and physiological amyloids. (a) Aβ40 fibril from AD patient brain. Adapted with permission from ref 184 Copyright 2019, Springer Nature. (b) Aβ42 fibrils from brain associated with sporadic (type I) and familial AD and other conditions (type II). Adapted with permission from ref 185 Copyright 2022, The American Association for the Advancement of Science. (c) Paired helical filaments (PHFs) and straight filaments (SFs) of tau from AD brain. Adapted with permission from ref 186 Copyright 2017, Springer Nature. (d) Patient derived α-synuclein filament with Lewy fold relevant to PD, PDD and DLB. Adapted with permission from ref 187 Copyright 2022, Springer Nature. (e) Vascular AA amyloidosis patient derived fibrils. Comparison of vascular and glomerular AA fibril structures. Adapted with permission from ref 188 Copyright 2022, Springer Nature. (f) Transthyretin fibril from hereditary Val30Met ATTR amyloidosis. Adapted with permission from ref 189 Copyright 2019, Springer Nature. (g) FapC cross-β fibril from *Pseudomonas* sp. UK4. Adapted with permission from ref 121 Copyright 2025, John Wiley and Sons. (h) Receptor interacting protein kinase-3 RHIM containing C-terminal domain (RIPK3-CTD) fibrils in right- and left-hand twists. Adapted from ref 190 Copyright 2021, National Academy of Sciences. (i) Structure of Orb2 filaments, a synaptic translation regulator. Adapted with permission from ref 18 Copyright 2020, The American Association for the Advancement of Science. (j) Cross–β structures for the amphibian antimicrobial peptides uperin 3.5 and (k) aurein 3.3. Panels (k) and (j) were adapted with permission from ref 120 Copyright 2022, Springer Nature. Notably, uperin 3.5 can adopt a cross–α structure in the presence of lipids. Dominant handedness has been reported for specific fibril polymorphs, where Aβ40 and α-synuclein filaments predominantly adopted right-handed twists and Aβ42, PHF tau, SF tau, serum amyloid A1 filaments in vascular AA amyloidosis, and transthyretin filaments in ATTR amyloidosis exhibited left-handed twists (a-f), but polymorphism and sample preparation can influence the observed twist direction. FapC and Orb2 adopt left-handed twists, and RIPK3-CTD adopts both left- and right-handed twists (g-i). These functional amyloids demonstrate species-specific utility including biofilm stabilization for FapC, activation of the innate immune response for RIPK3-CTD, and memory formation in Drosophila for Orb2.

Beyond pathology, many organisms leverage functional amyloids to carry out intracellular and extracellular biological processes. Amyloids have codified roles for chemical storage, structure, information, loss-of-function, and signaling/gain-of-function (see Table 1 for examples of each function).^15^ Moreover, functional amyloids are prevalent in bacteria as biofilms.^16^ It was recently shown that biofilm-associated proteins (BAPs) with amyloidogenic properties in the gut microbiota can assemble into amyloid-like fibrils, and these BAP-derived amyloids can induce α-synuclein aggregation and recapitulate pathological features of Parkinson’s disease when inoculated in the wild-type mouse brain.^17^ As such, coaggregation and cross-seeding are important considerations when considering amyloids for human use. We also highlight the synaptic translation regulator Orb2, which is important for maintenance and recall of memory in flies and forms an amyloid with a core enriched in polar hydrophilic residues, whereas pathological amyloids are often enriched in hydrophobic amino acids, illustrating that amyloids in this case could be a stable but flexible substrate for memory.^18^

Protein aggregation depends not simply on the quantity of hydrophobic or hydrophilic amino acids in the monomer, but rather on their specific arrangement and distribution along the protein backbone, for example into aggregation prone regions (APRs). The specific solvent environment (aqueous or membrane) is also important, and, collectively, these features can favor the formation of intermolecular β-sheets and adoption of the amyloid state. In particular, APRs are ∼ 5–15 residue long stretches of a peptide sequence that have the tendency to self-associate into the intermolecular cross-β structure, with rich backbone hydrogen bonding of the APRs from layer to layer in the cross-β spine. These layers often tightly interdigitate with neighboring layers to from steric zippers.^19^ In addition to hydrophobic content in APRs, side chain size, charge, and β-sheet propensity within these regions are also highly relevant to aggregation propensity, as is the solvent accessibility of the APRs.^20–23^

Collectively, amyloid formation occurs in pathology and physiology. Numerous naturally occurring or rationally designed peptides and proteins can adopt the amyloid state for diverse applications that are the focus of Section 2 below. Readers with a detailed understanding of the amyloid state may wish to proceed to Section 2, as we provide an overview of the biophysics, thermodynamics, and kinetics underpinning the amyloid state in the remainder of Section 1.

### 1.2 The Amyloid State

Many amyloids are comprised of 2–4 protofilaments that twist helically or laterally associate to produce higher order fibrils.^24^ The signature core-β core of amyloid fibrils consists of stacked β-strands arranged noncovalently and perpendicular to the fibril axis, forming a network of extensive hydrogen bonding between strands that contributes to the remarkable stability of the amyloid state (Figure 4), yielding strengths and stiffnesses (e.g., Young’s modulus) on the order of steel and silk, respectively,^25^ and rendering amyloidogenic monomers as promising building blocks for synthetic biological materials.^26^

**Figure 4:**
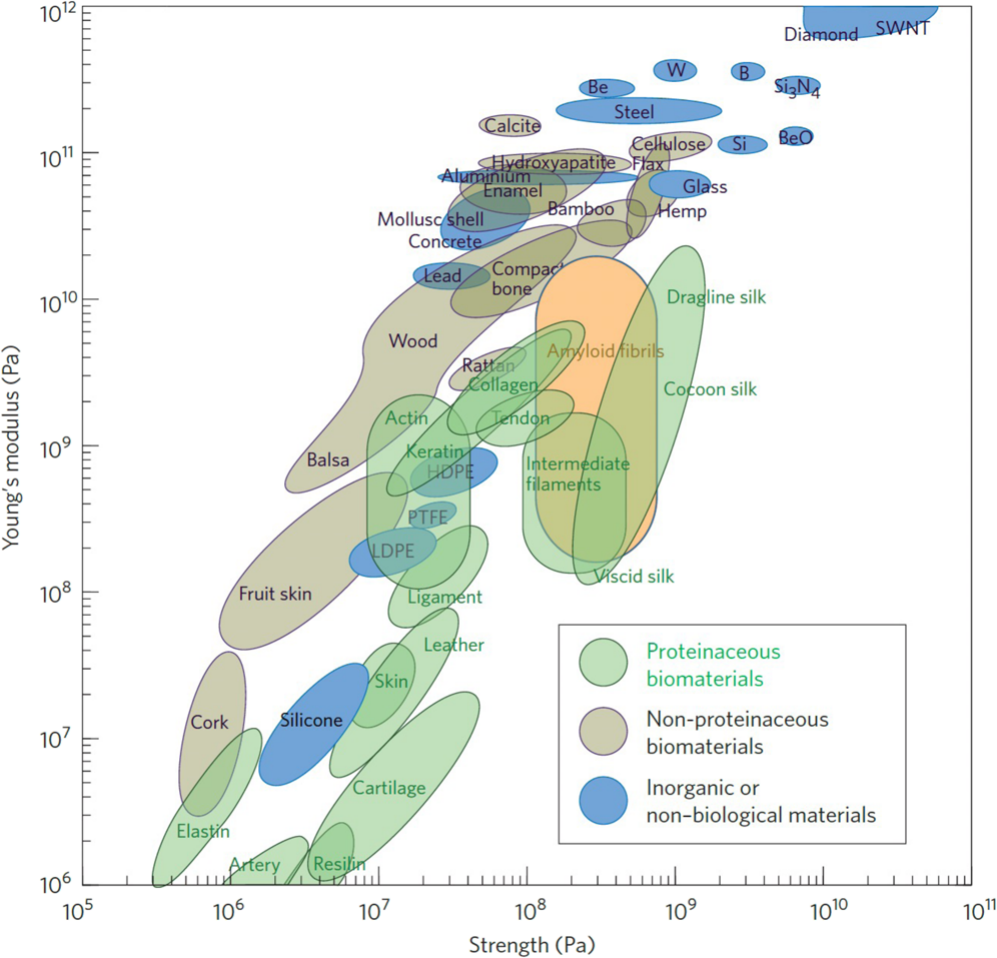
A comparison of the mechanical properties of amyloid fibrils to biological and inorganic materials. Young’s modulus (stiffness) versus strength for proteinaceous biomaterials (green), nonproteinaceous biomaterials (gray), and inorganic or nonbiological materials (blue). The range for amyloid fibrils is shown in orange. Reproduced with permission from ref 191 Copyright 2011, Springer Nature Limited.

The thread-like amyloid fibrils are relatively thin and long, often ranging in diameter from ∼ 6–12 nm^27^ and with lengths up to several micrometers,^28^ where these properties are influenced heavily by solution conditions and the peptide under investigation.^1^ Amyloid fibrils are recognized for their shared properties, in particular their cross-β structure,^1^ X-ray diffraction pattern,^29^ and hallmarked signatures by solid-state nuclear magnetic resonance spectroscopy.^30^ They also have common fibrillar morphologies as observed by multiple types of microscopy, specific tinctorial properties, high thermodynamic stabilities giving them resistance to denaturation and proteolysis, and, in the case of pathological amyloids, cytotoxicity (see Table 2 for further details).^1,2^ We refer to ref 31 for further fibril detection methods and ref 1 for the interplay between fibrils and protein misfolded oligomers in the induction and propagation of cytotoxicity in pathology.

**Table 2:**
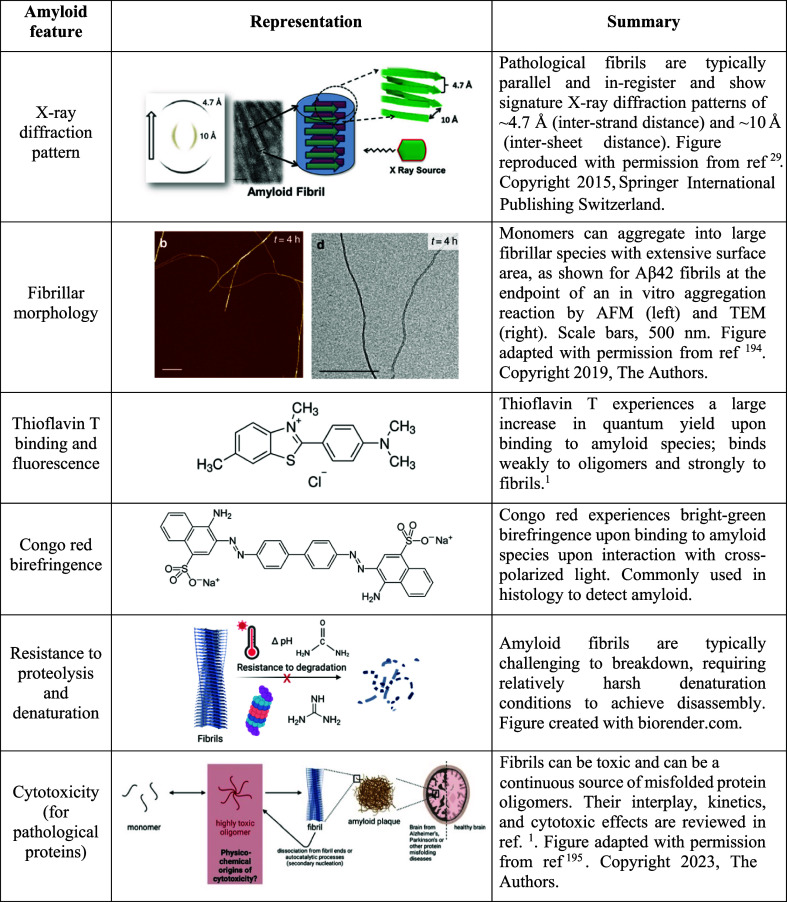
Hallmark Features of the Amyloid State^193,194^

Despite the vast differences in the monomeric peptides and proteins that compose amyloids, as well as their unique atomic fibrillar structures and differential capacities for supramolecular assembly (Figure 3), amyloids often have rather conserved structural and mechanical properties. The cross-β motif yields viscoelastic nanostructures with extensive mechanical strength. Amyloid fibril strength can be similar to that of steel and their stiffness comparable to that of silk (see Figure 4 for comparisons to additional proteinaceous or non-proteinaceous biomaterials, as well as inorganic or non-biological materials).^32,33,191^ The combined relatively high tensile strength, Young’s modulus, and shear modulus can make fibrils resistant to breakage; however, fibril fragmentation can nonetheless occur, for example with shaking in vitro or prion diseases in vivo.^1^ Amyloids are generally also resistant to heat, pH, and proteases,^24^ especially in the case of pathological fibrils. The mechanical properties and templating capabilities that make amyloids notoriously challenging to treat in afflicted patients have the potential to facilitate the creation of useful nanomaterials to solve a variety of modern problems. Since amyloid fibril formation is a noncovalent assembly process, it is reversible; however, in many pathological states, amyloid dissociation is orders of magnitude slower than its formation, rendering the process essentially irreversible. Reversibility therefore appears critical to the continuation of healthy rather than pathological states, as exemplified by functional amyloids, and is therefore also an important consideration in the design of amyloid-based biomaterials.^34^

### 1.3 Thermodynamics and Kinetics Regulating the Amyloid State

Native unstructured and structured proteins alike can access the amyloid state through partially folded intermediates. Conversion from the native to amyloid state is a complex and dynamical process, requiring passage through several intermediates of aggregation. We refer herein to the soluble and multimeric aggregates that are intermediates to fibril formation as misfolded protein oligomers, which are considered by many to be critically important cytotoxic protein aggregates in many protein misfolding diseases.^1^ In the free energy landscape of protein folding and aggregation, which is sensitive to protein sequence and solution conditions, this gives rise to a multitude of conformations that are accessible as intermediates between the unfolded state and funneling down toward the native state through intramolecular contacts or the amyloid state via intermolecular contacts (Figure 5a). The amyloid state represents a minimum in the free energy landscape, rendering it potentially even more stable than the native state.^1,2,12^ Protein engineering experiments have illustrated that mutating individual residues can stabilize high-energy transition states and partially folded intermediates, therein potentially facilitating or attenuating access to the amyloid state.^12^ This exemplifies that protein sequence can be tuned to drive aggregation toward or away from specific states. Advances in artificial intelligence and machine learning may enable scientists to rationally design peptide sequences to produce amyloid biomaterials with tuned thermodynamic and kinetic assembly principles and negligible toxicity, including upon their degradation. Care should be taken if designing or changing amyloidogenic sequences, and protein aggregates should be disposed of according to approved protocols that meet current safety standards.

**Figure 5:**
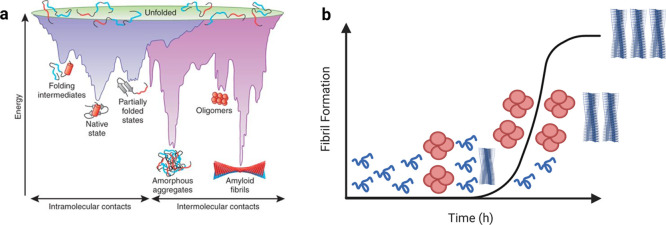
Thermodynamics and kinetics of protein aggregation. (a) A schematic representation of an energy landscape for protein folding (blue) and aggregation (purple). A multitude of conformations can funnel toward the native state via intramolecular contacts, or toward the formation of amyloid fibrils via intermolecular contacts. Reproduced with permission from ref 200 Copyright 2009, Springer Nature America, Inc. (b) Schematic of the fibril formation process in vitro, which can manifest through a variety of techniques as a sigmoidal curve with a lag phase characterized predominantly by monomer nucleation into early aggregates, a growth phase dominated by secondary processes (microscopic steps that require the presence of fibrils), and a plateau phase where free monomer is depleted in the reaction. For pathogenic proteins, oligomeric aggregates that are highly cytotoxic can form. The concentration of oligomers typically peaks near the half-time of the aggregation reaction, as shown. Created with biorender.com. This process is readily tracked using modern biophysical techniques, to include Thioflavin T fluorescence.^1^ Analytical tools enable the determination of microscopic mechanisms governing protein aggregation from macroscopic observables.^192^

Protein aggregation can be monitored macroscopically (Figure 5b). We refer to refs 1 and 2 for detailed reviews on the fundamental microscopic steps governing protein assembly, including primary nucleation (*k*_*n*_), elongation (*k*_+_), oligomer association and dissociation, surface-catalyzed secondary nucleation (*k*_2_), fragmentation (*k*_–_), and others, noting that these steps and their relative rates can range from rate limiting to negligible depending on the protein being studied and its solution conditions. The quiescent aggregation rate for α-synuclein, for example, can prominently increase at mildly acidic pH values as a function of enhanced secondary nucleation.^4^ Reaction rates also depend on the concentrations of monomers and fibrils present.

Thermodynamic and kinetic studies have shown that mutating the sequence of a protein, changing solution conditions, or adding small and large molecules, such as inhibitors, off-pathway species stabilizers, or molecular chaperones, can dramatically change protein aggregation mechanisms and associated rate constants.^3,4,35–40^ Comparing toxic pathological amyloids to functional ones, it is hypothesized that a combination of structural differences, regulation of the aggregation reaction away from secondary processes (namely secondary nucleation and fragmentation, that accelerate toxic oligomer formation), and very fast fibril formation for functional amyloids evolved in some concerted part toward preventing the accumulation of oligomeric aggregates in the controlled assembly of functional amyloids with minimized toxicity.^15^ The templating abilities of amyloid fibrils can be particularly useful in creating amyloid materials in bulk, wherein the introduction of fibril seeds at the start of an aggregation reaction can rapidly accelerate monomer conversion to a fibrillar structure.^1^ Collectively, these thermodynamic and kinetic principles outline the range of features that can be manipulated to change and direct the fibril formation process.

## 2 Amyloid Fibril-Based Materials Science Applications

Amyloidogenic proteins can be attractive building blocks for various biological and material applications, owing to their ability to form spontaneously for a broad range of proteins and peptides under a variety of solution conditions.^41–44^ In this section, we overview amyloid fibril-based material applications, including hydrogels and aerogels, tissue engineering, drug delivery, antimicrobials, detection and purification of specific analytes, food science and protein-based packaging, chemical catalysis, and bioelectronics.

### 2.1 Hydrogels and Aerogels

With their high stiffness and tunable chemical functionality, amyloid fibrils (or fibers) offer versatile building blocks to assemble 3-dimensional hydrogel networks, or aerogels with solvent removal (Figure 6). Hydrogels are soft, malleable materials formed from a network of solid phase material suspended in water which is the majority phase, up to greater than 90%.^45^ The high-water content of hydrogels allows for the encapsulation and diffusion of various molecules.^46^ Amyloid hydrogels and aerogels provide an alternative material strategy to conventional polymers, and enable a wide range of biomedical and biotechnology applications to include tissue engineering, implantable medical device coatings, drug delivery, filtration, and chemical catalysis.^47–54^ While protein-based amyloid fiber hydrogels may not currently offer the same controlled material and mechanical properties as synthetic polymer hydrogels due to fiber polymorphism, they are able to replicate biological environments with excellent biocompatibility making them potentially well-suited for biomedical applications.^52,55,56^ Moreover, amyloid-based hydrogels can be exploited through molecular design and tailored to specific applications.

**Figure 6:**
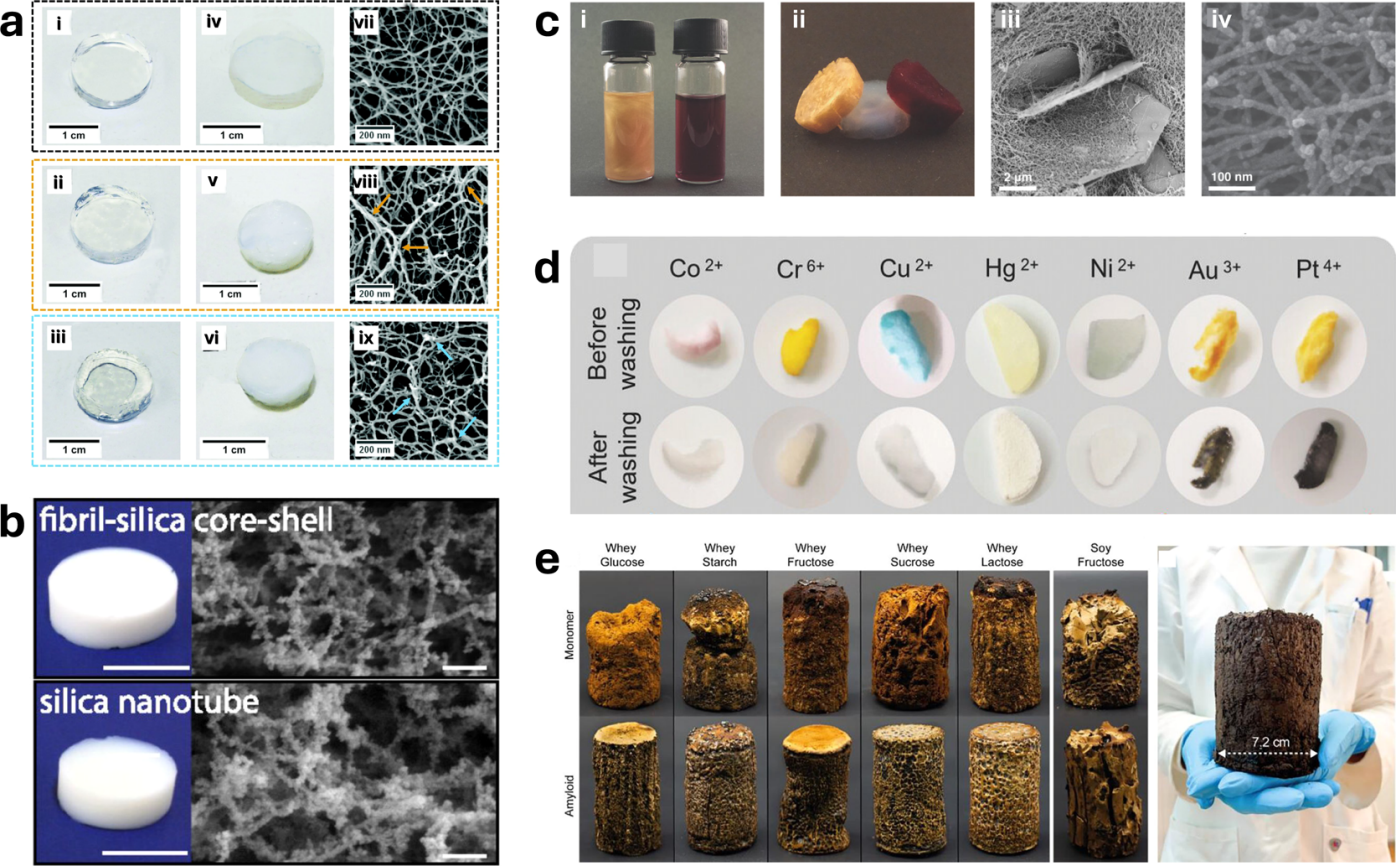
Hydrogel and aerogel applications of amyloids. (a) Hydrogels and aerogels made from pure βLG amyloid fibrils, of βLG/Gellan Gum hybrid networks and of βLG/κ-carrageenan hybrid materials, respectively. (i–iii) Pictures of the pure/hybrid hydrogels. (iv–vi) Pictures of the pure/hybrid aerogels. (vii–ix) Microscopic characterization of the formed aerogels through Scanning Electron Microscopy (SEM) imaging. In panels viii and ix, the colored arrows highlight local bundles. Adapted with permission from ref 68 Copyright 2021, Royal Society of Chemistry. (b) Photos and SEM images of the fibril–silica core–shell aerogel (upper) and silica nanotube aerogel (lower). The silica nanotube aerogel was generated by calcining the fibril–silica aerogel at ∼700 °C for 2 h. (Scales bars in photos and SEM images: 1 cm and 200 nm, respectively.) Reproduced with permission from ref 76 Copyright 2019, National Academy of Sciences. (c) Photographs of (i) amyloid fibril gold microcrystal (left) and nanoparticle (right) dispersions and (ii) resulting gold microcrystal (left) and nanoparticle (right) aerogels shown together with a pure amyloid fibril aerogel (center). Scanning electron micrographs (SEM) of (iii) amyloid fibril gold microcrystal and (iv) gold nanoparticle aerogels. Adapted with permission from ref 71 Copyright 2017, John Wiley and Sons. (d) The washing effect of 0.1 M EDTA solution on heavy metal ion loaded amyloid/ZIF-8 hybrid aerogel. Adapted with permission from ref 77 Copyright 2018, the Authors. (e) Carbon aerogels produced from different carbohydrates and proteins, such as whey and soy. (right) Upscaled carbon aerogel from amyloid whey and lactose. Reproduced with permission from ref 81 Copyright 2022, the Authors.

Amyloid hydrogels are synthesized through protein self-assembly and physical entanglement of fibers and may be further reinforced through covalent or ionic cross-linking.^45^ The protein solution temperature, pH, and ionic strength may also be used to facilitate nanofiber self-assembly and hydrogel formation.^55^ The diversity of proteins used for amyloid fiber formation, the ability to chemically modify and functionalize the fibers, and facile incorporation of other biological or inorganic materials provides a vast array of possible materials. As an example of hydrogel formation from a protein solution, Yan and colleagues demonstrated a thermoreversible hydrogel by heating a solution of hen egg white lysozyme (HEWL) in the presence of dithiothreitol to form an entangled network of 1 μm long fibrils with diameters 4–6 nm.^48^ Hydrogels may be formed from physically entangled amyloid fibers, as demonstrated by Su et al. using β-lactoglobulin fibrils with atomically dispersed iron atoms coordinated to nitrogen sites for oral administration to catalyze ethanol oxidation to acetic acid.^57^ Physically entangled self-healing hydrogels have also been formed from 5% and 10% bovine serum albumin solutions using a tris­(2-carboxyethyl)­phosphine to reduce disulfide bonds to induce fibril and gel formation.^58^ Physically entangled hydrogels have also been formed from the peptide sequence RSAIEDLLFDKV from common coronaviruses demonstrating the range of amyloid fibers possible for gel formation, as well as extending the range of pH tunable protein stability.^59^

Hydrogels have a three-dimensional hydrophilic network wherein different types of therapeutics can be encapsulated. Nagai and colleagues demonstrated self-assembling fibril and hydrogel formation from the acetyl-(Arg-Ala-Asp-Ala)_4_-CONH_2_ peptide,^46^ and later demonstrated the hydrogel’s encapsulation of a trypsin inhibitor, lysozyme, immunoglobulin, and bovine serum albumin (BSA). Diffusing protein size and hydrogel density, with gel pore sizes ranging from 5 to 200 nm in diameter, were found to influence diffusivity showing hydrogel design as a means to control protein release kinetics.^60^ In another study, organophosphate hydrolase (OPH), a chemical detoxification and bioremediation agent, was covalently cross-linked with glutaraldehyde and immobilized with bovine insulin fibrils that resulted in a 300% enhanced thermal stability of the enzyme at higher temperatures of 40, 45, and 50 °C, relative to its free counterpart.^61^ Amyloid fibril hydrogels can also immobilize enzymes, providing an environment to stabilize and enhance their catalytic properties. β-Lactoglobulin amyloid fibril hydrogels were used to coimmobilize seven Calvin Cycle enzymes – RuBisCO, 3-phosphoglyceric phosphokinase, α-glycerophosphate dehydrogenase, triosephosphate isomerase, glyceraldehyde 3-phosphate dehydrogenase, glycerol 3-phosphate oxidase, and catalase – converting carbon dioxide to fructose.^49^ Simple phenylalanine amino acid amyloid fibril hydrogels have also been shown to exhibit a high surface elastic modulus up to 30 GPa and a storage modulus of 0.7 MPa at concentrations of 0.24 M.^62^ In an effort to address the challenge of modifying hydrogel surfaces in an aqueous environment, amyloid-like nanofilms formed from phase transitioned human lactoferrin were shown to adhere to a range of hydrogels including poly­(2-hydroxyethyl methacrylate) and poly­(acrylic acid), poly­(vinyl alcohol) (PVA), agarose, and polyacrylamide.^63^

Composite amyloid hydrogels with more than one solid material component have been shown to enhance gel material properties, such as stiffness and energy absorption. To enhance the fibrillogenesis process, How et al. prepared hydrogels from β-lactoglobulin combined with BSA by a cold-set gelation process.^64^ BSA improved the fibrillar network and protected the hydrogel from loss of fibrils due to changes in pH and also increased the solid phase surface homogeneity of hydrogel with a correlation between the number of fibrils and the in vitro release rate of riboflavin. In another study on β-lactoglobulin hydrogels, Shen et al. prepared colloids with calcium ions and calcium nanoparticles to cross-link fibrils, thereby resulting in a gel at neutral pH with robust stiffness and strength properties.^65^ This hybrid hydrogel exhibited a storage modulus two orders of magnitude higher than previously demonstrated Ca^2+^ induced amyloid hydrogels and close to the stiffness of human tissues.^66,67^ In addition to using proteins, nanoparticles, and divalent ions to reinforce amyloid fibril networks, Usuelli et al. diffused low acetylated gellan gum and κ-carrageenan polysaccharides into the β-lactoglobulin (βLG) fibrillar networks, thereby preserving the surface functionality and enhanced mechanical properties of the resulting hydrogels and aerogels (Figure 6a).^68^ A modular approach to programmable composite amyloid fibril-based hydrogels with multifunctionality was demonstrated by Wang et al. with a triple fusion protein consisting of a soft amyloid core to mediate fibrillation, a mussel protein for adhesion, and a protein tag to functionally decorate the fibrils with proteins and molecules of interest.^69^ This modular approach was used to immobilize enzymes, coat 2-dimensional substrates, and form hydrogels for HEK 293T cell adhesion and growth.

To expand the range of potential applications, hydrogels have also been demonstrated as aerogels with the liquid phase replaced by gas through supercritical drying, which minimizes structural damage to the solid phase network and maintains the shape and volume of the original gel.^70^ The linear geometry of amyloid fibers and ability to assemble them into networks makes amyloid hydrogels an appealing synthesis platform to achieve 3-dimensional inorganic/organic materials (Figure 6c).^71^ Protein functional groups on the amyloid network fibrils are able to nucleate and stabilize a wide range of inorganic nanoparticles. In particular, β-lactoglobulin amyloid gels have been used to template silver and gold nanoparticles for potential antibacterial, sensor, and catalysis applications.^71–73^ Other metals, such as platinum^74^ and iron,^75^ as well as calcium nanoparticles,^65^ have been templated on amyloid fibers to form functional aerogels. Another example of an organic–inorganic amyloid-based aerogel is a fibril and silica core–shell nanostructure prepared by mixing β-lactoglobulin or lysozyme fibrils with a tetraethyl orthosilicate precursor resulting in aerogels with an approximately 20 GPa stiffness similar to some inorganic metal and alloy materials (Figure 6b).^76^ This composite core resulted in mechanically stable aerogels and stretchable double network hydrogels, which when calcined retained the aerogel monolith shape and microstructure with a 993 m^2^/g specific surface area. In the same study, the β-lactoglobulin networks were also infiltrated with polyacrylamide to form a double network composite that enhanced mechanical stiffness demonstrating the versatility of amyloid network structures for aerogel synthesis. β-Lactoglobulin amyloid fibrils networks have also been used as templates to prepare metal–organic framework zeolitic imidazolate framework-8 hybrid aerogels (Figure 6d).^77^ The hybrid aerogel was highly stable in water, homogeneous, and ultralight, demonstrating excellent adsorption toward heavy metals such as Hg^2+^ (1376 mg g^–1^), Au^3+^ (503 mg g^–1^) and Pb^2+^ (318 mg g^–1^) compared to amyloid fibril aerogels without ZIF-8.

Another interesting use of amyloid fibers for aerogel synthesis is through hydrothermal carbonization (HTC), where carbonaceous material is generated in water at elevated pressures and low temperatures between 180 and 250 °C.^78^ The resulting carbon aerogels belong to a category of materials synthesized from biomass.^79^ However, traditional active forms of carbons are known to perform poorly with heavy metals, and have high energy demands during synthesis at high temperatures.^80^ Carbon aerogels synthesized via HTC not only requires lower energy consumption compared to traditional activated carbon, but the carbon particle size, surface structure, and porosity can be tailored in aqueous environments. Peydayesh and colleagues fabricated hydrogels and aerogels using HTC of proteins in the amyloid and monomeric forms with different carbohydrates, such as glucose, fructose, starch, lactose and sucrose (Figure 6e).^81^ The amyloid based aerogels exhibited high stability, order, and uniform structure relative to the monomer-based aerogels and selectively adsorbed gold, iron, silver, and platinum with an application of whey amyloid fiber aerogels to selectively capture gold from electronic waste.^82^ We also note that multiple studies have used amyloid fibrils to form hydrogels and aerogels to capture carbon dioxide.^52,83–85^

### 2.2 Tissue Engineering

Amyloid fibrils can mimic biological properties of extracellular matrix (ECM), which is an integral component of organs and tissues promoting cell adhesion, migration, and differentiation.^86,87^ Biomimetic, self-assembling peptides offer great potential for low-cost and high purity scaffolds for in vitro and in vivo cell growth.^88^ Different types of amyloid fibrils, independent of their sequence, composition, and in the absence of integrin binding sites, have been shown to facilitate cell adhesion and integrin signaling (Figure 7a).^86^ These unique ECM-like properties and the generic cell adhesivity of amyloids make them pragmatic biomaterials for tissue engineering.^86,89,90^

**Figure 7:**
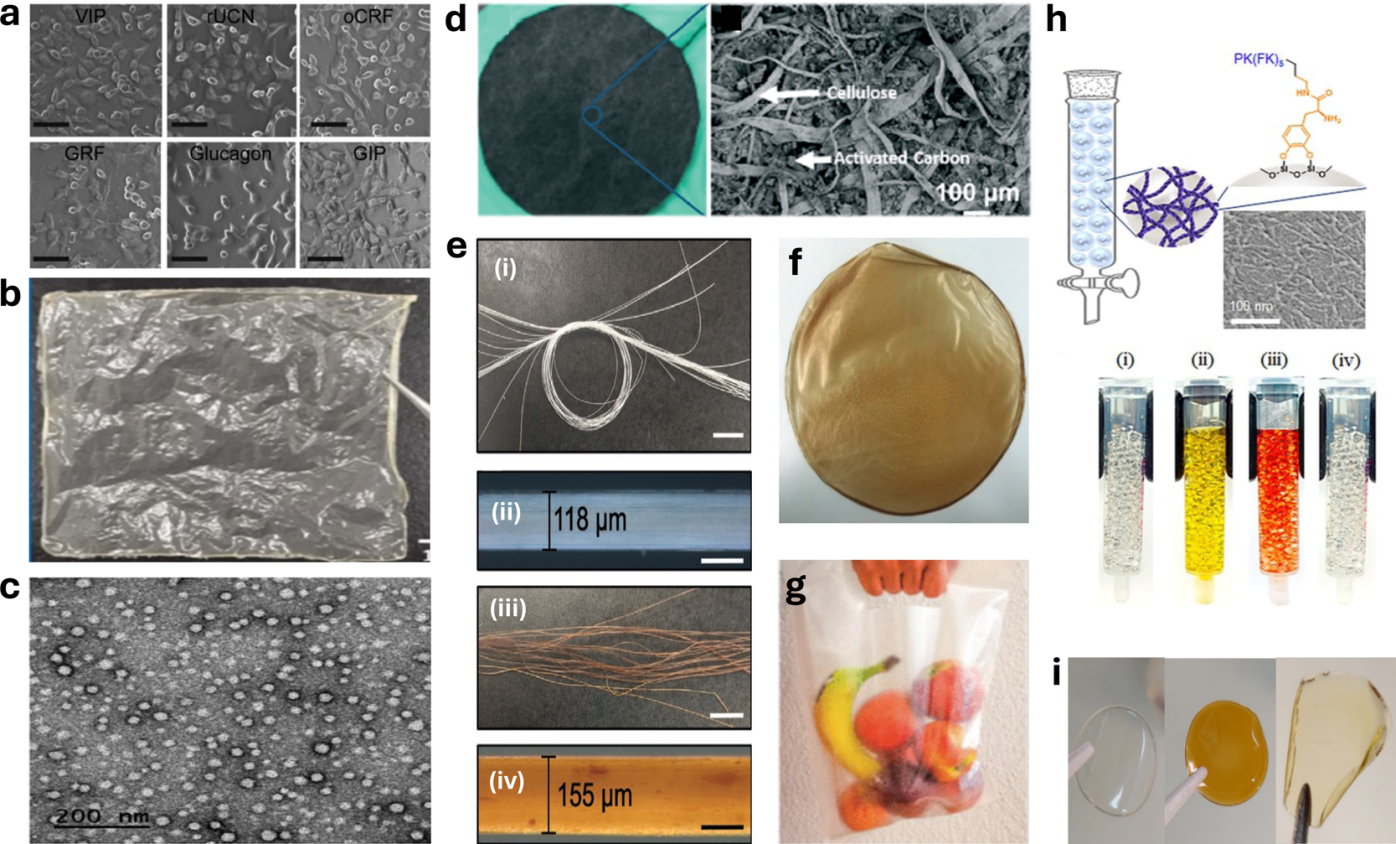
Tissue engineering, filtration, food packaging, drug delivery, biosensing, catalytic, and bioelectronic applications of amyloids. (a) Cell adhesion on amyloid fibrils for tissue engineering. SH-SY5Y were cultured on various protein/peptide amyloid fibrils showing cell adhesion on the substrate. Scale bars, 100 μm. Adapted with permission from ref 86 Copyright 2016, Elsevier. (b) Physical appearance of carboxymethyl cellulose (CMC)/whey protein isolate amyloid fibril (WPI-AF) membrane for drug delivery. Adapted with permission from ref 103 Copyright 2023, Multidisciplinary Digital Publishing Institute. (c) High magnification transmission electron microscopy image of Sup35-5aa-DHFR-Z biocompatible amyloid-like oligomeric nanoparticles for drug delivery. Scale bar, 200 nm. Reproduced with permission from ref 106 Copyright 2020, American Chemical Society. (d) Photos and SEM images of a cropped β-lactoglobulin-carbon membrane for water filtration. Reproduced with permission from ref 132 Copyright 2020, Royal Society of Chemistry. (e) Physical appearance of spun wire made from gelatin-β-lactoglobulin amyloid fibrils (i and ii, scale bars 1 cm and 100 mm, respectively) and spun wire hybrid made from gelatin-β-lactoglobulin amyloid fibrils/Fe_3_O_4_ (iii and iv, scale bars 1 cm and 100 mm, respectively) for actuator or sensor applications. Adapted with permission from ref 135 Copyright 2020, John Wiley and Sons, Inc. (f) Photo of a bioplastic film produced with rapeseed cake amyloid fibrils combined with a biodegradable polymer, (poly)­vinyl alcohol, and a plasticizer, glycerol. Reproduced with permission from ref 141 Copyright 2023, John Wiley and Sons, Inc. (g) Amyloid fibril-biodegradable polymer blends formed from whey protein, engineered into a film to produce a bioplastic bag for packaging. Reproduced with permission from ref 142 Copyright 2021, American Chemical Society. (h) SEM image of PFK_DOPA_-silica bead catalyst (2 mm bead diameter, coated with 3 mM PFK_DOPA_) (Scale bar, 100 nm) with photos showing the hydrolysis of nitrocefin at various stages (i–iv) using a glass column filled with the PFK_DOPA_-silica bead catalyst. Reproduced with permission from ref 171 Copyright 2025, Springer Nature. (i) Physical appearance of mixed feather keratin amyloid fibril membranes in different stages of production ultimately to be used as proton-conductive membranes for fuel cells. Reproduced with permission from ref 176 Copyright 2023, American Chemical Society.

In the 1990s, Zhang and colleagues demonstrated that amphiphilic protein sequences (RAD16, EAK16) formed 3D porous matrices that facilitated the attachment of a range of cell types.^91^ In another study using amyloid fibrils for cell adhesion, Kasai and colleagues used the A208 peptide obtained from mouse laminin chain α1 (2097–2108), coupled with arginine-glycine-aspartic acid (RGD) or glycine-arginine-glycine-aspartic acid-serine (GRGDS), to promote human fibroblast cell adhesion on amyloid scaffolds.^92^ The protein sequences were selected based on fibronectin cell adhesion active sites and the authors observed that the human fibroblast cells adhered and spread well on the engineered fibrils. To demonstrate the use of amyloid fiber network topography for cellular attachment, Reynolds and colleagues deposited hen egg white lysozyme fibers on 2-dimensional mica substrates, and then subsequently deposited a plasma polymer film on top.^88^ With this approach, the authors were able to control the surface and binding chemistry through the deposited polymer film and investigate the influence of the amyloid fiber surface topology on fibroblast cell binding and spreading. Cells were observed to more significantly bind to the polymer surface with the amyloid surface topography compared to flat polymer surfaces. As an extension of this work, Reynolds again deposited HEWL amyloid fibril networks on flat mica substrates with variable widths and surface coverage.^93^ The authors observed that high areal surface coverages from larger sized amyloid fibrils resulted in favorable cell adhesion and spreading. As an extension of 2D engineered surfaces with amyloid fibers for cell attachment, Das et al. mixed amyloid fibers and graphene sheets and cast onto a 2D substrate to form a composite film with oriented fibrils.^94^ The films with aligned amyloid fibrils on graphene presented a periodic trough and crest surface without the use of expensive lithographic or etching techniques. This surface topology mediated the attachment, polarization, and differentiation of neural precursor cells to form polarized or elongated morphologies with neurite projections. Such functionalized protein sequences and amyloid fiber nanocomposites can serve toward engineering scaffolds that promote interactions with a broad range of cell types.

More recently, Shaw and colleagues demonstrated hydrogels formed from pentapeptides with good biocompatibility and thixotropy for skin wound healing by promoting cell migration, adhesion, along with extracellular matrix remodeling.^95^ Polyphenols such as tannic acid, epigallocatechin gallate, and catechin were added to lysozyme to form highly adhesive hydrogels demonstrated to serve as cell scaffolds for HeLa and mouse fibroblast cells.^96^ To address the challenge of microbial infection in tissue engineering, Chen and colleagues developed a composite antibacterial hydrogel comprised of lysozyme amyloid fibrils cross-linked to polyethylene glycol (PEG) that offers both injectability and shape compatibility.^97^ This approach leveraged the innate antimicrobial properties of lysozyme fibers and mitigated the swelling of the PEG gel phase and proved effective against both Gram-negative *E. coli* and Gram-positive *S. aureus* bacteria. Mukherjee et al. also demonstrated an antibacterial hydrogel formed from proteins with modified Aβ42 peptide cores that exhibited antibacterial properties against *S. aureus* and was able to rescue dermal fibroblasts from cocultured bacterial damage.^98^ The antibacterial hydrogels also promoted the healing of diabetic wounds from Gram-negative *P. aeruginosa* and Gram-positive MRSA-infections by reducing inflammatory response and enhancing angiogenesis.

Amyloid fibers have also been used to engineer bone tissue. Li and colleagues used a vacuum-assisted filtration to combine one-dimensional amyloid fibril assembly and two-dimensional hydroxyapatite (HA) and brushite platelets for creating biomimetic structures with similar material properties to cancellous bone.^90^ The authors further demonstrated the growth of human trabecular bone-derived osteoblast cells cultured on the amyloid-HA composites. Leveraging the abundance and hierarchical porosity of plants, Li et al. coated decellularized plant material with nano amyloids and hydroxyapatite nanocrystals as a scaffold to promote bone regeneration.^99^ Another interesting composite materials approach for vascularized bone regeneration was demonstrated by Yang et al., who used HEWL to prepare an injectable amyloid fibril, clay nanosheet, DNA hydrogel with a dense network of amyloid fibrils that demonstrated thixotropic behavior and low cytotoxicity.^100^ The gel components synergistically enhanced the hydrogel mechanical strength and conferred shear-thinning, injectability, self-healing, and 3D printable properties. QK peptide release from amyloid fibrils facilitated human umbilical vein endothelial cells tube formation and migration, while Si^4+^ and Mg^2+^ ion release from the clay component phase promoted osteogenic differentiation of bone marrow mesenchymal stem cells.

### 2.3 Drug Delivery

Drug delivery research has been advancing toward approaches for the effective, controlled (tissue or cell specific) delivery of therapeutics to maximize efficacy and minimize off-target effects, including for various routes of administration such as the oral, buccal, sublingual, nasal, ophthalmic, transdermal, subcutaneous, and others. Drug solubility, absorption, bioavailability, and biocompatibility are major challenges in engineering drug delivery systems. The material properties and potential biocompatibility of amyloid fibrils make them promising agents in drug delivery. Amyloid fibrils can be functionalized with surface modifications,^101,102^ they can have useful biodegradability and biocompatibility profiles, and their stability in varied environments allows them to be utilized as repositories of molecules or of active proteins or peptides in therapeutics.

Amyloid-based membranes encompassing carboxymethyl cellulose and whey protein isolate amyloid fibril were synthesized by cross-linking with glutaraldehyde, thereby forming composites with an interconnected network. Following cross-linking, the hybrid membranes were used as a vehicle to release cationic and hydrophobic drugs. Moreover, results indicated that the drug release was dependent upon the drug–membrane interactions, highlighting the potential of amyloids in transdermal drug delivery (Figure 7b).^103^ A composite material approach was shown by Chang et al. by grafting whey protein isolate fibrils with poly (acrylic acid) for the diffusion and swelling based drug delivery of oppositely charged molecules metformin and vitamin C.^104^ Trusova et al. combined phospholipids and amyloid fiber hydrogels for a composite lipid vesicle-amyloid hydrogel drug delivery carrier using combinations of phosphatidylcholine and cardiolipin to encapsulate lysozyme and bovine serum albumin amyloid fibrils for doxorubicin and europium coordination complex delivery with implications for increased hydrophilic drug loading efficiency.^51^

Liquid crystalline mesophases (LCMs) drug delivery systems have also been investigated for topical routes of administration for drugs with low solubility and bioavailability and rapid metabolism. Resveratrol, a natural polyphenol with anti-inflammatory activity, has been captured into LCMs containing phytantriol and β-lactoglobulin fibrils. LCMs are known for self-assembling properties and are capable of solubilizing lipophilic and hydrophilic drugs, facilitating drug stability and controlled drug release. The addition of these fibrils improved the bioadhesivity of the LCMs topicals and the controlled release of resveratrol in in vitro and ex vivo assays, and resveratrol was retained by epidermis and dermis.^105^ Interestingly, the interaction between fibrils and cellular surfaces via focal adhesion machinery has been reported to facilitate integrin-mediated cell adhesions similar to that of the ECM proteins.^86,105^ These results suggest that amyloid fibrils as scaffolding proteins have potential in nontoxic drug delivery systems that target cell-specificity and controlled release.

Wang et al. utilized the soft amyloid core (SAC) of yeast amyloidogenic sequence to develop a module that can be loaded with drugs and monoclonal antibodies for cell-specific targeted drug delivery (Figure 7c).^106^ The SAC residues of Sup35 in yeast were fused to *E. coli* dihydrofolate reductase enzyme with an 8-residue-long linker consisting of serine glycine repeats. The authors showed that the linker length regulated the assembly of catalytic fibril nanoparticles that were stable, homogeneous, and nontoxic. The nanoparticles were then fused with antibody-binding domain (Z-domain) resulting in multifunctional nanoparticles. When loaded with methotrexate, this system preferentially targeted cancer cells.^106^ Ovalbumin-based nanofibrils also have shown the potential to aid in the sustained and targeted delivery of chemotherapeutics and immunotherapeutics.^107^

### 2.4 Purification and Detection

Water purification is enabled by filters that are inexpensive to produce and reusable. Amyloids have been engineered to purify water from contaminants, including metal ions, radioactive material, fluoride, per- and polyfluoroalkyl substances (PFAS) and organic pollutants from water sources. Amyloid fibrils can also be functionalized to detect changing environmental conditions or capture macromolecules or cells (Table 3).

**Table 3:** Examples of Amyloid-Based Filtration Membranes for Water Purification

type of material	constituent peptide or protein	advantageous material properties	filtered substance	ref
filtration membrane	β-lactoglobulin amyloid and activated carbon	abundant, inexpensive, capable of filtering a wide range of contaminants	heavy metals, radioactive material, per- and polyfluoroalkyl substances	^129,132,133^
	β-lactoglobulin amyloid with zirconium oxide and activated carbon		fluoride	^130^
	β-lactoglobulin aerogel		organic contaminants	^131^
	lysozyme		microplastics	^134^

β-Lactoglobulin amyloid fibrils combined with activated carbon produced mechanically strong composite materials, where the amyloid fibrils were the main component of the two responsible for capturing heavy metals. β-Lactoglobulin alone filtered the heavy metal ions, potassium dicyanoaurate, mercury­(II) chloride, lead­(IV) acetate, and disodium tetrachloropalladate, but when combined with activated carbon, the composite material filtered out the metal ions more than the amyloid alone, reducing the initial ppm metal ion concentration of each sample by three to four orders of magnitude.^129^ Moreover, when β-lactoglobulin amyloid fibrils coated with zirconium oxide nanoparticles were mixed with activated carbon, the resulting hybrid membrane was able to filter fluoride from water with a 95.5% removal efficiency.^130^ The presence of other anionic specific species like SO_4_^2–^, Cl^–^, and NO_3_^–^ did not greatly influence the ability of the hybrid membrane to filter fluoride. The hybrid membrane had a higher selectivity for fluoride compared to commercial resins specifically made to filter fluoride, and it maintained a high efficiency of filtration over a wide range of pH of 2 to 10.^130^ In another instance, amyloid fibril aerogels prepared from 2 wt % aqueous β-lactoglobulin amyloid fibrils were used to filter representative organic contaminants like bentazone (pesticides), bisphenol A (phenolics), and ibuprofen (pharmaceuticals), resulting in removal efficiencies of 92%, 78%, and 98%, respectively.^131^

The β-lactoglobulin amyloid fibril and activated carbon composite material also filtered uranyl acetate and phosphorus-32 with high efficiency, reducing the concentration of the radioactive species in water by 3 orders of magnitude. The presence of both amyloid fibrils and activated carbon were important, the prior for higher filtration efficiency and the latter for improved processing flow rates.^129^ Other radioactive material has been filtered using 10 wt % β-lactoglobulin amyloid fibrils mixed with activated carbon (Figure 7d).^132^ The hybrid membrane was highly efficient, filtering technetium-99 and iodine-123 by 99.999%, 99.822% for gallium-68, 99.95% for lutetium-177, and 99.995% for iodine-131. This technology proved to be scalable, showing the possibility of converting large volumes of liquid radioactive waste into low volumes of solid radioactive waste.^132^

A study compared the filtration efficiencies of pure β-lactoglobulin fibril membranes, β-lactoglobulin fibril membranes on cellulose acetate, and amyloid-carbon hybrid membranes and their ability to remove PFAS of varying sizes ranging from 214 to 714 Da. The filtration efficiencies of the pure amyloid fibril membranes and the amyloid fibril membranes with the cellulose acetate improved as the PFAS molecular weight increased, with the greatest filtration efficiencies seen from 414 to 714 Da. The amyloid–carbon-based hybrid membrane of β-lactoglobulin with activated carbon was able to remove a range of long-chain and short-chain PFASs, offering complete removal of PFASs with ≥4 perfluorinated carbon atoms in the molecular structure and a removal efficiency of low molecular weight perfluorobutanoic acid (3 perfluorinated carbon atoms) exceeding 96%.^133^ Lysozyme amyloid fibrils were also prepared as a flocculant to aggregate small particles like microplastics.^134^

Amyloid fibrils can be used for developing biosensing materials to detect biomolecules or to detect cells through their adhesion, as well as to measure colorimetric and fluorescent responses.^135–140^ Lee et al. created electronic textiles from a combination of β-lactoglobulin fibrils, reduced graphene oxide, and cotton yarn for the purpose of detecting hazardous gases like nitrogen dioxide, ammonia, formaldehyde, hydrogen, or sulfur dioxide.^138^ The use of β-lactoglobulin amyloid fibrils takes advantage of the fibrils enhanced adhesion to graphene, acting as an adhesion promotor between the graphene and cotton yarn, improving active sites for high sensitivity and selectivity.^138^ Highlighting the versatility of β-lactoglobulin amyloid fibrils, Lutz-Bueno et al. combined gelatin with β-lactoglobulin amyloid fibrils to produce spun wires, utilizing gelatin’s plasticity alongside the rigidity and multifunctional capabilities of amyloid fibrils for use in actuator or sensor applications (Figure 7e).^135^ Amyloid-based colorimetric materials were created using α-synuclein and 10,12-pentacosadiynoic acid (PCDA) by coincubating both components and then exposing them to UV light. The α-synuclein fibril-PCDA sensor changes color from blue to red when subjected to heat, pH variations, or ethanol.^139^ Another type of stimuli-responsive sensor consists of amyloid curli fibers (CsgA) fused with a pH-sensitive protein called pHuji (CsgA-pHuji).^140^ These complexes can respond to changes in pH and effectively differentiate between alkaline and acidic solutions. In both cases, the sensors possess inherent resistance to harsh conditions due to the mechanical strength of the fibrils. The yeast protein Sup35 was genetically fused with a Z-domain, a designed analogue of the B domain from *S. aureus* protein A that binds with high affinity to the Fc region of antibodies. These biocompatible, antibody-decorated multivalent nanorods can identify and enhance interactions between different cell types, including T lymphocytes and tumor cells.^137^

### 2.5 Protein-Based Packaging and Food Science

The use of nonbiodegradable single-use plastics to package food has long-term environmental and health implications that may be mitigated by development of protein-based biomaterials. Amyloids offer highly ordered β-sheet structures that afford properties traditional biomaterials may not, including high tensile strength, decreased water permeability due to hydrophobic β-sheet stacks, increased stability at a range of temperatures, and possible UV resistance.^47^ Largescale development of biodegradable plastics remains a challenge due to the costs associated with production. As such, food waste products, such as whey, soy, and gluten, have been proposed as a mechanism of amyloid bioplastic development. Bagnani et al. utilized rapeseed cakes, a waste biproduct of canola oil manufacturing, to develop a proof-of-concept bioplastic extraction process, where napin and cruciferin amyloid isolates derived from isoelectric point precipitation showed the highest water stability and contact angle compared to other methodologies (Figure 7f).^141^

Despite challenges, amyloids remain potentially advantageous for food packaging due to combinatorial design strategies that allow for tunability toward desirable qualities. β-Lactoglobulin has been combined with PVA to generate a transparent bioplastic with high resistance to mechanical stress and stable resistance to water vapor.^142^ In this study, scalability was also assessed with methylcellulose (MC), which was more sustainable than PVA but showed reduced stability in the presence of water and lower elasticity (Figure 7g).^142^ Zein protein-derived amyloid fibrils have been developed into sustainable bioplastic films for food packaging.^143^ Liu et al. demonstrated that soy or whey protein amyloid isolates combined with vanillin had antibacterial properties while retaining the shape of the film.^144^ Furthermore, work has been conducted to hybridize plant derived protein fibrils with polysaccharides to improve strength, toughness, and gas selectivity, ultimately reducing food spoilage, and improving tensile strength.^145^

While the amyloid state is classically associated with pathology, numerous food proteins are reported to assemble into amyloid fibrils.^31^ Briefly, amyloids derived from animals include β-lactoglobulin and α-lactalbumin from whey, caseins, ovalbumin, lysozyme, ovotransferrin, bovine serum albumin, hemoglobin, and ferritin. Plant amyloids include glutelin, prolamin, and gliadin from wheat, α-zein, albumin and globulin from rice. Additional detail and more exhaustive lists can be found in refs 31 (for amyloids) and^146^ (for many food proteins). Various physical, chemical, and biological properties of amyloids can be leveraged into food-related applications, including heat resistance, reduced allergenicity, and use as gelators, thickening agents, foams, and emulsion stabilizers.^31,147–151^

Naturally occurring amyloids are being explored to improve food manufacturing and nutritional outcomes. Initial studies suggest that soy amyloids may be implemented as scaffolds for cellular deposits, initially with C2C12 myogenic mice cells, a methodology that may prove useful for meat cultivation.^152^ Another potential advantage of amyloids is in supplementing nutritional needs, as suggested by Shen et al., where β-lactoglobulin amyloids were used as iron nanoparticle carriers for supplementation in iron depleted rats.^75^ Two soy protein amyloid nanoparticles also demonstrated the ability to reduce iron­(III) to the more bioavailable form iron­(II), which could help prevent iron deficiency anemia.^153^ While the use of amyloids for nutritional and recycling purposes hold promise remains a valuable area of interest, questions remain around the breakdown of amyloids in digestion, as well as cost to benefit analyses to further improve utility.^152,154^

### 2.6 Chemical Catalysis

Enzymes possess remarkable catalytic properties due to their unique three-dimensional structures. However, they can denature when exposed to extreme temperatures, pH changes, high salt concentrations, or specific chemicals, disrupting the bonds that maintain their structure and functionality. Current research aims to design amyloid fibrils that can act as catalysts while retaining their activity under harsh conditions that typically impair enzymes. In vitro studies show that naturally occurring amyloid fibrils (amyloid-β, α-synuclein, and glucagon) can catalyze biological reactions,^155–158^ suggesting the potential for synthetic amyloids to mimic enzyme-like catalysis with improved structural stability and versatility (Table 4).

**Table 4:** Examples of Natural and Synthetic Amyloid Fibril Catalysts

constituent peptide or protein	application	catalyst description	catalyzed reaction	ref
amyloid-β	natural catalyst	amyloid-β (1–42)	hydrolysis of standard substrates, oxidation of neurotransmitters	^195^
glucagon		glucagon (1–29)	esterolysis, lipid hydrolysis, and dephosphorylation	^158,196^
α-synuclein		α-synuclein (wild-type and 1–119)	esterolysis, dephosphorylation, metabolite composition alteration	^197,198^
Im-KLVFFAL-NH_2_	synthetic catalyst	catalytic dyads (imidazoles and lysines) interspersed in the chiral amyloid binding grooves	enantioselective catalysis	^160^
Ac-^16^RLVFFA^22^L-NH_2_, Ac-^16^BLVFFA^22^L-NH_2_		short amyloid-based nanotubes and NaBH_4_	ester reduction	^159^
Ac-NFGAIL-NH_2_		catalysis driven by amyloid–substrate complex	amine nucleophilic reactions	^161^
PABPN1(N-(+7)Ala)-CspB		fusion of one domain protein to amyloidogenic fragment	hydrolysis of peptidoglycan	^162^
bovine insulin amyloid fibrils cross-linked to glucose oxidase		fully formed fibrils modified through cross-linking with enzyme	oxidation of glucose	^163^
Ac-LHLHLQL-NH_2_ + Zn^2+^, Ac-LHLHLRL-NH_2_ + Zn^2+^, Ac-IHIHIQI-NH_2_ + Zn^2+^		heptapeptides: alternating apolar residues for assembly, other residues for catalysis/ion binding	Zn^2+^-dependent esterolysis	^164,165^
Ac-NADFDGDQMAVHV-NH_2_ + Mg^2+^/Mn^2+^		conserved polymerase sequence followed by amyloid-prone region	Mg^2+^/Mn^2+^-dependent hydrolysis of phosphoanhydride bonds	^166^
Ac-SDIDVFI-NH_2_ + Mn^2+^		Mn^2+^ coordinates H_2_O with phosphate and aspartate of fibril surface	Mn^2+^-dependent hydrolysis of phosphoanhydride bonds	^168^
TTR(105–115) with ε-azido-Lys mod at 108		FeMC6*a covalently bonded to peptide	oxidation of the model substrate ABTS	^170^
Ac-PK(FK)_5_P–NH_2_ variants		alternating lysine/phenylalanine β-sheet-forming sequence	hydrolysis of β-lactam antibiotics	^171^

Amyloid fibrils generated from the hexapeptide Ac-^16^KLVFF^21^A-NH_2_, from the nucleating core of Aβ42, have proven versatile in catalyzing reactions,^159^ including enantioselective hydrolysis, owing to the combined effort of the imidazole and lysine at the C-terminal end of the heptapeptide sequence Im-KLVFFAL-NH_2_ (cat-KL).^160^ Recently, Chatterjee et al., using synthetically derived Ac-^16^RLVFFA^22^L-NH_2_, created short amyloid-based nanotubes (ARG-16) with surface exposed cationic residues for colocalization of substrates, along with the weak hydride transfer sodium borohydride cofactor, to reduce esters to alcohols in water.^159^ Building on this initial effort, a more effective catalytic short amyloid-based nanotube (BET-16) was produced by replacing the arginine residue of ARG-16 with a quaternized glycine (betaine trimethylammonium salt, B), resulting in the Ac-^16^BLVFFA^22^L-NH_2_ sequence. BET-16 had a 60.5 ± 16% conversion of esters to alcohol compared to the 27.5 ± 4% of ARG-16, maintaining a product yield of 42.3 ± 7% after three consecutive cycles of being recycled, demonstrating their reusability.^159^ The islet amyloid polypeptide-derived sequence Ac-NFGAIL-NH_2_ (NL6) was also able to promote nucleophilic amine reactions like acylation, arylation, cyclization and alkylation, in acidic buffer, owing to the carbonyl oxygen of the Phe–Gly amide bond playing a key role in activating the substrate amine through hydrogen bonding.^161^

Other catalytic amyloids have been developed with the amino acid sequences necessary to create the core β-sheet characteristic of amyloids and the catalytic domains necessary to promote specific chemical reactions. Synthetic amyloid fibrils have been created by (1) producing a globular enzyme of interest fused to an amyloid-forming sequence,^162^ (2) the addition of enzymatically active proteins through cross-linking or chemical modification of already formed fibrils,^163^ and (3) designing small peptides that require the binding of divalent metals to aggregate and possess catalytic activity.^164–169^

A recent method to produce a synthetic amyloid catalyst was to identify a self-assembling amyloidogenic peptide like human transthyretin (TTR(105–115)) and install an analog of the Mimochrome family of artificial metalloenzymes through click chemistry with a ε-azido-Lys moiety substituted at position 108 of the peptide.^170^ In this study, the fibrils, deemed FeMC6*@fibrils, retained the morphology of the unfunctionalized peptide and the catalytic activity of the artificial enzyme, showing that the immobilization of the enzyme on amyloid fibrils can be an efficient strategy to protect catalysts from deactivation. Moreover, the FeMC6*@fibrils when deposited on polyvinylidene difluoride filter membranes, were able to go through 40 cycles of oxidation before losing their activity, showcasing the fibrils reusability and stability.^170^

Another recent methodology to create a synthetic amyloid catalyst is to use amphiphilic peptides rich in lysine residues capable of being nucleophiles in the hydrolysis of β-lactam antibiotics like penicillin and amoxicillin,^171^ similar to the lysine environment found on the alpha phenol-soluble modulins of functional bacterial amyloids secreted by *S. aureus*.^122^ The cationic Ac-PK­(FK)_5_P–NH_2_ variants, dubbed PFK amyloid fibrils, that were tethered to commercially available silica beads (PFK_DOPA_/bead) in a column-filter setup, degraded β-lactam antibiotics dissolved in water (Figure 7h), demonstrating good recyclability and efficacy.^171^

### 2.7 Bioelectronics

Due to the inherent properties of amyloids and their biodegradable nature, research on their use as bioelectronic components is ongoing.^172–177^ Shipps et al. measured charge transport by applying current to four different fibrils formed by the peptide segments with or without a metal cofactor: Zn^2+^-NNQQNY, GNNQQNY, Zn^2+^-GGVLVN, and KVQIINKKL through a four-electrode setup, noting that measuring proper protein conductivity has been hampered by artifacts due to large contact resistances between proteins and electrodes.^173^ Of the four fibrils, those formed from the Zn^2+^-NNQQNY peptide with metal cofactor had the highest conductivity of 3.5 ± 0.96 μS/cm, with the GNNQQNY peptide being 6-fold less in conductance, 0.59 ± 0.20 μS/cm, showing the combination of stacked tyrosines with a metal cofactor allowed the greatest fibril conductance.^173^ The group ultimately determined that electron transport was due to micrometer-long chains of stacked tyrosines through a multistep hopping mechanism. Their results also confirmed that contact resistance plays a significant role in measurements of protein conductivity, suggesting the need for a complementary electron-conducting scaffold between the electrodes and the protein.

To further exploit the electronic properties of amyloid fibrils, Kihal et al. sought to design biocompatible semiconductive materials through the creation of perylene diimide (PDI)-conjugated amyloid peptides.^175^ PDI has π-conjugated systems with semiconductor properties, but they are poorly water-soluble. By symmetrically linking PDI with a self-assembling amyloid peptide sequence of Ac-SNNFGAILSS-NH_2_, the central 20–29 region of the islet amyloid polypeptide, they were able to guide PDI organization into ordered conductive and biocompatible nanofilaments (PDI­[I_10_]_2_). In addition to the PDI molecule and the self-assembly peptide unit, a two-charged lysine sequence (KK) was added to enhance solubility, and a GSGS-tetrapeptide linker was also incorporated between the PDI and peptide to prevent interference with the peptide sequence’s aggregation into fibrils. High conductivity (I > 1.0 nA) was measured for the symmetric (PDI­[I_10_]_2_) peptide-based film at 10 V scanning voltage. These fibrils exhibited higher stability after application of higher voltages (±10 V) compared to fibrils assembled by the peptide sequence alone.^175^

Amyloids can specifically be used in battery and fuel cell applications. β-Lactoglobulin fibrils formed at pH 2 and at 90 °C can be vacuum-filtered on a glass-fiber separator (AF5@GF) for use in sodium batteries.^172^ The sodiophilic amyloid fibril homogenizes the electric field and Na-ion concentration in ester-based electrolytes, thereby inhibiting dendrite formation—a factor that has hindered the large-scale applications of sodium-based batteries. Sodium (anode) and Na_3_V_2_(PO_4_)_3_ (cathode) batteries with AF5@GF separator ran with 87.13% capacity retention after 1,000 cycles.^172^

Another example of an amyloid energy source is the use of keratin, an 18-amino acid peptide, extracted from reused industrial chicken feathers. This peptide is being transformed into fibrils and incorporated into a proton exchange membrane (Figure 7i), which selectively allows protons to pass between the electrodes of a fuel cell.^176^ To have these proton exchange membrane properties, the membrane-incorporated fibrils must go through an oxidative process to transform the thiol groups of the cysteine side chains of keratin into sulfonic acid groups.^176^ The ion exchange capacity increases from 0.18 ± 0.03 to 0.84 ± 0.03 mequiv g^–1^ when the neutral thiols are converted into negatively charged sulfonic acids. When incorporated into a commercial fuel cell setup, the keratin fibril membrane, with hydrogen at the anode and air at the cathode, was able to transfer protons and help the system generate power to turn on red and white LED lamps.^176^

### 2.8 Antimicrobials

The discovery, development, and ultimate usage of antimicrobial peptides (AMPs) is a growing field that has become inextricably linked to the amyloid state. The mechanisms of action between AMPs vary greatly based on structure, but fibrils have demonstrated abilities to cause innate immune stimulation, direct pore formation, and even sequestration of bacteria through fibril nanonets.^108,109^ Historically, the innate immune peptides LL-37, human defensin 6 (HD6), porcine derived protegrin-1 (PG-1), and lysozyme have demonstrated fibrillar properties that can be leveraged in de novo machine learning peptide design and screen to specific antimicrobial properties.^110–114^ Focusing on recent applications, Salinas et al. have shown that the AMP uperin 3.5, derived from the *Uperoleia mjobergii* Australian toadlet, forms cross-β fibrils. However, this structure is driven toward an antiparallel cross-α amyloid fibril when exposed to Gram-negative bacteria lipids resulting in pore formation in *Micrococcus luteus* (*M. luteus*), suggesting that the amyloid state and chameleon secondary structure are key to regulating antibacterial properties (Figure 8a).^115–118^ Further work from Ragonis-Bachar et al. suggested that a bioinformatic approach could be implemented based off known AMP sequences to identify those that may be fibril-forming. This study designed and assessed 14 fibril-forming peptides displaying a variety of properties, including time dependent fibril-formation, α-helical conformational shifts in the presence of lipid membranes, cytotoxicity, as well as variations in minimal inhibitory concentrations (MIC) values against *M. luteus* ranging from 1.3 μM to more than 250 μM, providing evidence that fibrillar AMPs vary in potential mechanisms of antimicrobial intervention.^119^ This study included aurein 3.3, and has since been elaborated on Bucker et al., who have discerned the cross-β structure of this fibril forming AMP and uperin 3.5 (Figure 3j,k).^120^

**Figure 8:**
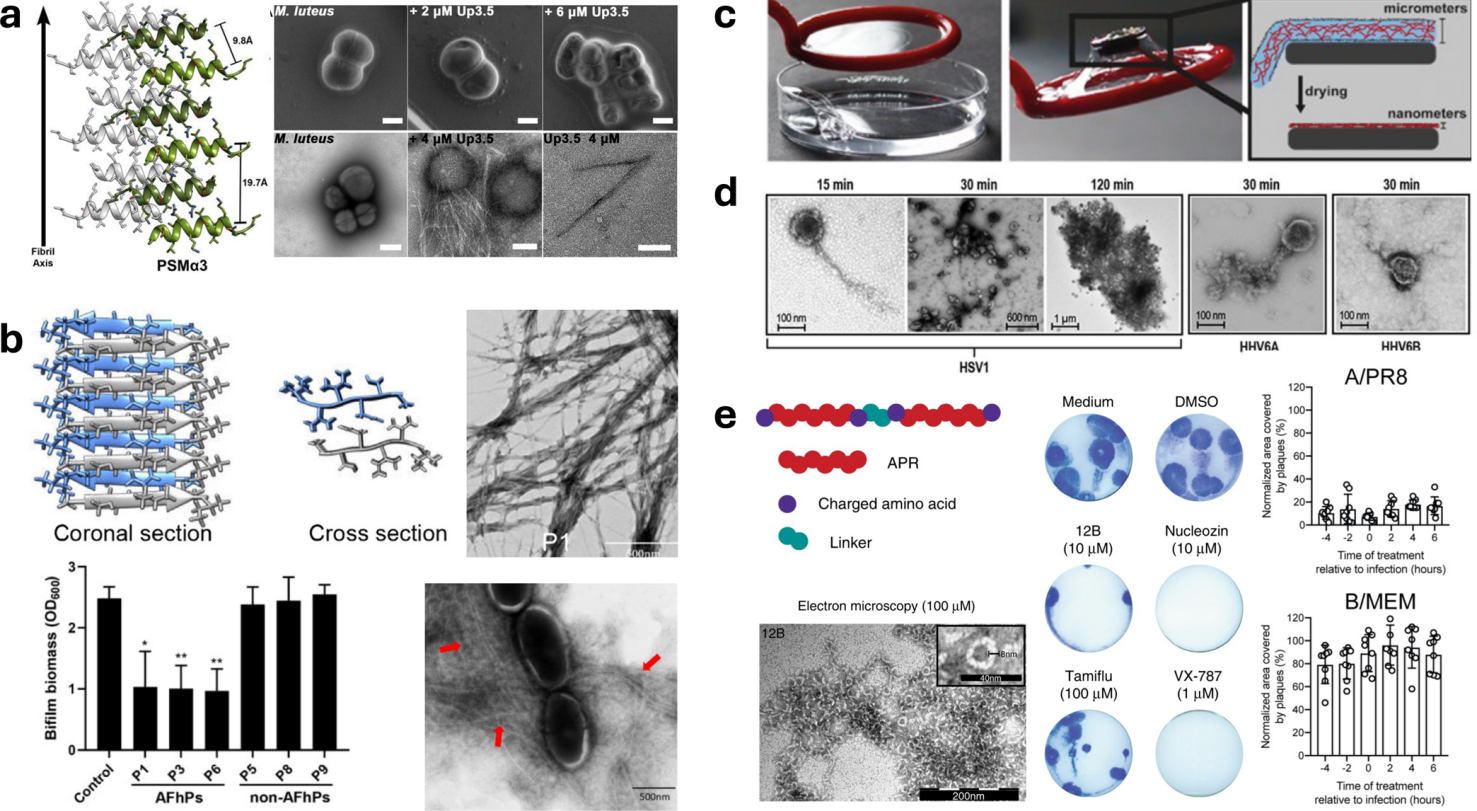
Antimicrobial peptides known to form amyloid structures and current utilization for antibacterial and antiviral purposes. (a) AMP uperin 3.5 displays a cross α-helical stack formation. *M. luteus* treated with uperin 3.5 in the presence of cells (bottom panel) results in amyloid formation as compared to without cells (top panel). Adapted with permission from ref 118 Copyright 2021, National Academy of Sciences. (b) Coronal and cross section of hexapeptide structure demonstrating parallel β-sheets. Sample hexapeptide 1 showing amyloid fibril formation in acidic solution. The effect of amyloid-forming hexapeptides (AFhPs) on biofilm biomass of *S. mutans* as compared with non-AFhPs. TEM images of AFhPs agglutinating to *S. mutans*. Adapted with permission from ref 123 Copyright 2020, John Wiley and Sons. (c) Physical appearance of hen egg white lysozyme amyloid fibril antimicrobial film. Reproduced with permission from ref 112 Copyright 2023, Royal Society of Chemistry. (d) HSV-1, human herpesvirus 6A (HHV6A), and human herpesvirus 6B (HHV6B) incubated in the presence of H4-Aβ42 at varying time points demonstrating aggregation around virions. Adapted with permission from ref 126 Copyright 2018, Elsevier. (e) Graphic depicting the design of antiviral peptides and the appropriate APR regions, connected with charged amino acids and a linker. Cryo-TEM demonstrating that the designed peptide forms amyloid-like structures. Comparative plaque sizes of IAV infection with 12B (developed peptide) and other antivirals. Comparative analysis of normalized area covered by plaques from IAV (A/PR8) and IAB (B/MEM) viruses as a function of treatment time with 12B, highlighting the specificity of the developed peptide for IAV. Adapted from ref 128 Copyright 2020, Springer Nature.

It is also widely reported that bacteria utilize, as demonstrated with the *fap* system in *Pseudomonas*, amyloids for the support of biofilm formation and structural support, offering practical tools for studying the natural utilization of fibrils for long-term pro-survival mechanisms (Figure 3g).^121^ This also holds in the context of understanding and combating antibiotic resistance, as demonstrated by *S. aureus*, where amyloid secretion led to the hydrolysis of amoxicillin and penicillin and survival of bacterial species.^122^ Chen et al. developed a library of 13 amyloid-forming hexapeptides that did not demonstrate direct microbial death but instead resulted in agglutination on the surface of *S. aurueus*, sequestering bacteria and suggesting a multitude of mechanisms for antimicrobial-amyloid development and potential interference with biofilm formation (Figure 8b).^123^ Moreover, a recent report in *E. coli* found Agp protein activates Bab protein through amyloid templating upon phage infection, making Bap a lethal effector that disrupts bacterial membrane integrity resulting in cell death and hindered phage replication.^124^ *E. coli* have also been observed to resist *B. bacterivorous* infection by producing curli fibers composed of oligomers of the functional amyloid protein CsgA.^125^ Additionally, hen egg white lysozyme has also been developed into amyloid fibril antimicrobial films (Figure 8c).^112^

One proposed mechanism to combat viral infection is to utilize aggregation prone AMPs for direct binding. Amyloid-β, particularly in the context of herpes simplex virus 1 (HSV-1), is reported to result in antiviral outcomes via virion sequestration, suggesting certain viral infections may be drivers of neurodegenerative disease by the promotion of amyloid formation as a mechanism for innate immune antimicrobial activity (Figure 8d).^126,127^ Michiels et al. developed two AMPs that display amyloid aggregation propensities with APRs against Zika (ZIKV) and influenza A. Through the usage of control peptides lacking the APR, they demonstrated that the amyloid state was necessary for inhibition of viral replication. This methodology may be implemented to develop highly selective amyloids for antiviral purposes, as shown in the lack of cross-reactivity demonstrated against influenza B and the ability to develop peptides across viral families, as with the flavivirus ZIKV (Figure 8e).^128^ Taken together, secondary structure of peptides is dependent upon environmental factors, driving the formation of α-helices and β-sheets, the conformation of which may be harnessed for improved stability, bioavailability, and act a reservoir between active and inactive states. Coupled with the flexible nature of AMPs sequences, the amyloid state lends itself to specific design strategies that further modulate immune stimulation, reduce host-toxicity, and potentially allow more targeted drug delivery.^118,119^

## 3 Current Challenges and Future Directions

Although amyloids were traditionally associated with neurodegenerative, systemic, and localized disease,^1,2^ they have more recently garnered considerable attention in the materials science space (Figure 2) owing to their unique physicochemical properties. In particular, the fibrillar form comprised of β-sheets can impart advantageous mechanical strength, chemical and thermal stability, and versatility. Moreover, amyloids can spontaneously self-assemble from their constituent precursor peptides or proteins, enabling the rapid bottom-up fabrication of nanostructures, which can minimize the need for harsh solvents or toxic reagents. Because they are formed from natural amino acids and proteins, many can be biocompatible and biodegradable.^142^ Amyloid precursors can be sourced from cheap source proteins and food industry byproducts, and their assembly can be inherently energy-efficient relative to petroleum-based materials.^142^ Owing to the fibrillar archetype, these nanostructures have characteristically high surface areas, which can be highly useful for increased adsorption, catalytic surfaces, and cellular interactions. Some are naturally antimicrobial or can be modified to promote this characteristic. As such, amyloid fibrils have appeared in various forms to address modern problems, including as diverse hydrogels and aerogels, conductive scaffolds for sensing, scaffolds for tissue engineering, coatings and films, antimicrobials, catalytic agents, biodegradable protein-based packaging materials, water purification, hybrid composites, in food science, and more (Figures 6–8).

The field of amyloid-based biotechnology is undergoing considerable growth. These large biomolecules are highly customizable and capable of forming different fibrillar polymorphs, to include novel and adapted primary sequences and post-translational modifications, co-factors present during the fibril formation process, heterotypic protofilaments or novel protofilament-protofilament interactions, postfibrillization modifications, through driving unique supramolecular assembly processes, and more. It is clear that solution conditions, such as pH, ionic strength, temperature, agitation/shaking, and the addition of discrete cofactors, can drive the formation of vastly differing amyloid structures and at differential rates of formation, further emphasizing the large optimization space to potentially make amyloids tailored to specific tasks. This is also a complex problem, as amino acids need to be selected that have the inherent ability to form an amyloid core in pragmatic time scales with low cost while preserving useful arrangements of amino acids on their surface to facilitate particular applications, such as surface catalysis. Specific properties can be optimized based on the desired outcome, such as favoring mechanical strength over flexibility or the exposure of specific catalytic sites. It is possible that artificial intelligence systems will enable the customization and optimization of amyloids for specific tasks once sufficient training data sets become available.

There are also clear challenges to overcome. While engineering costs can be relatively low, the formation of amyloids in bulk is inherently dependent upon protein sources that can be highly costly to use at scale, such as those from animal sources. These can come from synthetic biology to make recombinant protein at scale and specific feedstocks. The latter depends upon the industrial base, and we anticipate advances in synthetic biology will continue to make this technology more accessible in the future. While amyloids are highly optimizable, it will take considerable time and cost to improve them for specific applications, such as tuning their mechanical properties, length scales, time to manufacture, conversion efficiency from monomers to fibrils,^178^ optimizing solution conditions, finding the right cofactors, ensuring biocompatibility or resistance to breakdown, and much more. While evidence suggests nonpathogenic amyloids can be safe, further degradation and recyclability studies are warranted to ensure amyloids do not cause adverse ecological or medical consequences. Amyloids clearly vary in their inherent toxicity, ranging from considerably toxic for neurodegeneration-linked proteins like Aβ and α-synuclein, to nontoxic under tested conditions for instances of food-based amyloids like β-lactoglobulin and lysozyme.^179^ It has been shown for pathogenic fibrils that toxic oligomers can detach from fibril ends,^180^ prompting further research into the long-term stability and byproducts from the customizable fibrils described above. For biomedical amyloid technologies, it is important to consider that pathogenic amyloids have been observed to cross-seed with other proteins, for example Aβ42 and α-synuclein^181^ or sup35NM and Aβ42,^182^ prompting detailed coaggregation studies for biomedical devices containing amyloids in addition to assessing their immunoreactivity.^183^ Lastly, using the amyloid state for materials science and engineering is an emerging technology, and this form of a protein is classically associated with devastating diseases. The integration of amyloid materials science and engineering applications into society will depend in great part on both validating their safety and educating the end users. Despite these challenges, amyloids clearly hold promise to advance materials research to address diverse problems in modern society.

## 4 Conclusion

The unique structural properties and assembly mechanisms of amyloids, long associated with pathology, are being harnessed worldwide for diverse biotechnological applications. By examining the principles of amyloid formation in disease and physiology, this review highlights the potential of amyloid-based materials in areas ranging from drug delivery and tissue engineering to protein-based packaging, chemical catalysis, antimicrobials, and bioelectronics. As the field advances, significant opportunities and challenges remain in translating amyloid-based structures into practical, safe, and societally impactful technologies.

## Data Availability

No original data were created in this review.
